# The Naples prognosis score as an independent predictor of sepsis-associated acute kidney injury: a retrospective cohort study

**DOI:** 10.3389/fmed.2025.1671981

**Published:** 2026-02-02

**Authors:** Hao Hong, Jindan Kong, Yao Wei, Jun Jin, Xueke Liu

**Affiliations:** Department of Critical Care Medicine, The First Affiliated Hospital of Soochow University, Suzhou, China

**Keywords:** acute kidney injury, inflammatory, Naples prognosis score, nutrition, sepsis

## Abstract

**Objective:**

This study aimed to evaluate the predictive efficacy of the Naples prognosis score (NPS) in sepsis-associated acute kidney injury (SA-AKI) and explore its mechanistic link to renal injury, with the goal of providing a novel clinical tool for early SA-AKI risk assessment.

**Methods:**

One-way ANOVA was applied to variables exhibiting normal distribution and homo-geneous variance. Spearman analysis was performed to assess relationships. Non-normal distribution variables were analyzed using the rank sum test. Binary and ordered logistic regression analyses were conducted to evaluate independent relationships with SA-AKI. Receiver operating characteristic (ROC) curve was employed to determine diagnostic accuracy. The survival curve was plotted by Kaplan Meier.

**Results:**

The NPS score was significantly higher in the SA-AKI group than in the non-AKI group (*P* < 0.001). Multivariate logistic regression showed that NPS was an independent predictor of SA-AKI (OR = 11.777, *P* < 0.001), with an area under the ROC curve (AUC) of 0.855. Correlation analysis indicated positive associations of NPS with renal injury markers (urea nitrogen, serum creatinine, cystatin C) and negative associations with platelet count and low-density lipoprotein. Subgroup analyses demonstrated that NPS effectively predicted SA-AKI regardless of vasopressor use or continuous renal replacement therapy (CRRT), with AUC values of 0.898 in the vasopressor group and 0.866 in the non-CRRT group.

**Conclusion:**

Naples prognosis score serves as an independent predictor of SA-AKI, integrating inflammatory, nutritional, and metabolic markers to provide new insights into SA-AKI pathophysiology. Clinically, NPS offers a simple and feasible tool for early identification of high-risk SA-AKI patients, guiding personalized treatment strategies.

## 1 Introduction

Sepsis-associated acute kidney injury (SA-AKI) imposes a substantial burden on critical care medicine and contributes to a more higher mortality rate (1, 2). The pathophysiology of SA-AKI involves a complex interplay of microcirculatory dysfunction, immune dysregulation, and direct renal tubular injury, highlighting the need for comprehensive prognostic tools (3, 4). Current diagnostic markers, such as serum creatinine and urine output, suffer from inherent limitations, often failing to detect renal injury in its early stages (5). Organ dysfunction scores like SOFA, while widely used, lack renal specificity and may overlook subtle metabolic and inflammatory derangements (6).

The Naples prognostic score (NPS) is based on inflammatory markers and nutritional status. It was first proposed by Garzia et al. in a colorectal cancer study (7). To date, the prognostic significance of this score has been validated in various cancers: neutrophil-to-lymphocyte ratio (NLR), lymphocyte-to-monocyte ratio (LMR), serum albumin, and total cholesterol (8–10). NLR and LMR reflect the balance of inflammatory and immune responses, while albumin and cholesterol serve as surrogates for nutritional status and lipid metabolism—both critical in sepsis pathophysiology. Although NPS has demonstrated prognostic value in cancers, its role in SA-AKI remains uninvestigated.

This study aims to evaluate the predictive efficacy of NPS in SA-AKI and explore its mechanistic link to renal injury, with the goal of providing a novel clinical tool for early SA-AKI risk assessment.

## 2 Materials and methods

### 2.1 Study population

This single-center, retrospective observational study was conducted in the Intensive Care Unit of the First Affiliated Hospital of Suzhou University, Jiangsu Province, China, from May 2023 to May 2024. We estimated the sample size using G*Power software, accounting for a 10% loss to follow-up rate, resulting in more than 32 patients in each group. This study meets the inclusion criteria for the minimum sample size. The inclusion criteria encompassed adults who fulfilled the diagnostic criteria for sepsis and sepsis-associated acute kidney injury (SA-AKI) (11). Sepsis was defined according to the third international consensus definition of sepsis and septic shock (12). Acute kidney injury (AKI) was defined in accordance with the KDIGO criteria, utilizing daily serum creatinine measurements and hourly urine output data (13). In our study, we employed the SA-AKI definition proposed by the ADQI 28 Working Group to guide our analysis (14). Patients were classified as experiencing SA-AKI if their AKI was diagnosed between the first and seventh day following the diagnosis of sepsis, as per the established ADQI criteria. Conversely, if AKI manifested prior to the diagnosis of sepsis, the patient would not qualify as having SA-AKI under the defined parameters. The exclusion criteria for this study included: (1) Individuals with inherent coagulation dysfunction; (2) Patients with severe liver disease; (3) Patients with severe immunodeficiency; (4) Pregnant or lactating individuals; (5) Patients with ischemic heart disease and heart failure. The Research Ethics Committee of the First Affiliated Hospital of Soochow University approved the study protocol (no. 213). Exemption from informed consent has been explicitly approved in the ethics committee’s approval document, meeting the ethical requirements after de-identification of retrospective research data.

### 2.2 Laboratory measurements

To collect plasma, 3 ml of fasting venous blood was drawn in the early morning following admission and stored in EDTA anticoagulant tubes. The samples were maintained at room temperature for 20 min before being centrifuged at 3000 rpm and 4 °C for 10 min. Subsequently, 500 μl of the upper plasma layer was transferred to a 2 ml cryovial and frozen at −80 °C. Biochemical and conventional hematology indicators were measured using the OLYMPUS AU2700 automatic biochemical analyzer (Olympus, Japan) and the Beckman LH750 automatic hematology analyzer (Beckman, USA). Blood coagulation was assessed using the STA-R Evolution (STAGO, France). Cytokine levels were quantified via flow cytometry using the FACS Canto II (BD, USA).

### 2.3 Naples prognosis score

The NPS was calculated as the sum of the scores for four variables, in which an abnormal level of NLR (>2.96), and LMR (≤4.44), serum albumin (<40 g/L), and total cholesterol (≤4.65 mmol/L) was assigned a value of 1 (Table 1) (7).

**TABLE 1:** Naples prognosis score.

Points	NLR	LMR	Albumin (g/L)	Total Cholesterol (mmol/L)
0	≤2.96	>4.44	≥40	>4.65
1	>2.96	≤4.44	<40	≤4.65

NPS, Naples prognosis score; NLR, neutrophil-to-lymphocyte ratio; LMR, lymphocyte-to-monocyte ratio.

### 2.4 Statistical analysis

Statistical analyses were conducted using SPSS version 27.0, while GraphPad version 10.0 was employed for graphical plotting. Data are presented as mean ± SD, median, and interquartile range (IQR) for datasets that are normally and non-normally distributed. One-way ANOVA was performed for variables exhibiting normal distribution and homogeneous variance, whereas rank sum tests were utilized for non-normally distributed variables. Spearman correlation analysis was conducted to assess relationships among variables. Binary and ordered logistic regression analyses were executed to evaluate independent associations with SA-AKI. To account for confounding factors, multivariate logistic regression was employed to adjust for potential biases. The receiver operating characteristic (ROC) curve was utilized to determine diagnostic accuracy, and the Youden index was calculated to establish the optimal cutoff point of the ROC curve. *P*-values of <0.05 (*) and <0.001 (**) were deemed statistically significant. The survival curve was plotted by Kaplan Meier.

## 3 Results

### 3.1 Baseline characteristics

During the study period, 92 patients with sepsis resulting from bacterial infections were considered for participation. 11 participants were excluded, comprising (1) 5 patients lacking laboratory data and (2) 6 patients who had no serum creatinine measurements due to death on Day 1. Consequently, a total of 81 sepsis patients were included in the study, consisting of 37 patients with AKI and 44 without AKI. Compared to the non-AKI group, the AKI group exhibited significantly higher Apache II scores (*P* = 0.003), SOFA scores (*P* = 0.007), and rates of CRRT (*P* < 0.001). Additionally, the use of vasoactive medications was significantly more prevalent in the AKI group (*P* = 0.025).

Among endothelial biomarkers, platelet count (PLT) (*P* = 0.028) and Albumin (ALB) (*P* < 0.001) was significantly lower, while activated partial thromboplastin time (APTT) (*P* < 0.001), Triglycerides (TG) (*P* = 0.008), Interleukin-8 (IL-8), and interleukin-10 (IL-10) (*P* = 0.019, 0.029, *P* < 0.001, respectively) levels were significantly higher in the AKI group. Additionally, kidney-related indicators, including urea nitrogen (UREA) and cystatin C (CYSC) (*P* < 0.001 for both), were significantly higher (Table 2).

**TABLE 2:** Baseline characteristics of sepsis patients with and without AKI.

Variable	Sepsis (*N* = 44)	SA-AKI (*N* = 37)	*P*-value
Age, years	75.000 (56.500, 80.000)	70.000 (54.000, 78.000)	0.608
Height, cm	166.000 (160.000, 170.000)	170.000 (160.000, 172.000)	0.584
Weight, kg	65.000 (58.000, 73.000)	65.000 (60.000, 70.000)	0.826
Male sex	29.5%	24.3%	0.422
APACHE II	15.000 (12.000, 20.000)	22.000 (16.000, 26.000)	0.003
SOFA	5.000 (4.000, 8.000)	9.500 (6.000, 12.000)	0.007
WBC (×10^9^/L)	6.420 (4.710, 20.600)	14.275 (9.180, 22.373)	0.443
NLR	21.039 (15.160, 26.920)	22.387 (18.272, 26.502)	0.188
LMR	2.774 (2.120, 3.429)	2.877 (1.584, 4.170)	0.623
RBC (×10^12^/L)	3.540 (3.320, 3.820)	3.580 (3.000, 3.905)	0.528
PLT (×10^9^/L)	204.000 (136.000, 295.000)	138.500 (72.250, 200.250)	0.028
CRP (mg/L)	153.690 (48.430, 238.380)	228.495 (97.993, 294.130)	0.097
ALB (g/L)	36.200 (34.573, 37.827)	31.695 (30.171, 33.219)	<0.001
TC (mmol/L)	3.090 (2.470, 3.630)	2.900 (2.230, 3.500)	0.313
TG (mmol/L)	1.250 (0.910, 1.800)	1.660 (1.388, 2.456)	0.008
SCR (μmol/L)	113.675 (97.822, 129.580)	178.559 (155.700, 201.419)	<0.001
UREA (μmol/L)	9.600 (7.400, 14.400)	17.900 (12.850, 23.223)	<0.001
CYSC (mg/L)	1.230 (0.830, 2.010)	1.875 (1.458, 3.233)	<0.001
PT (sec)	15.700 (15.000, 17.600)	16.550 (14.675, 20.300)	0.391
APTT (sec)	42.200 (35.200, 50.500)	48.850 (42.175, 61.025)	<0.001
IL-6 (pg/ml)	28.930 (6.030, 118.950)	94.135 (22.623, 484.953)	0.058
IL-8 (pg/ml)	14.670 (1.930, 31.810)	37.245 (14.890, 106.458)	0.029
IL-10 (pg/ml)	1.900 (0.590, 7.140)	6.445 (3.008, 15.365)	<0.001
28-day mortality	13.6%	37.8%	<0.001
CRRT	6.8%	48.6%	<0.001
Vasopressors	27.3%	56.8%	0.025

AKI, acute kidney injury; SA-AKI, sepsis associated acute kidney injury; APACHE II,Acute Physiology and Chronic Health Evaluation II score; SOFA, Sequential Organ Failure Assessment; WBC, white blood cell; NLR, neutrophil-to-lymphocyte ratio; LMR, lymphocyte-to-monocyte ratio; ALB, albumin; RBC, red blood cell; PLT, platelet; CRP, C reactive protein; TC, total cholesterol; TG, triglyceride; SCR, serum creatinine; UREA, Uric Acid; CYSC, cystatin C; PT, Prothrombin time; APTT, activated partial thromboplastin time; CRRT, continuous renal replacement therapy. Continuous variables were expressed as the median (quartile), and categorical variables were expressed as percentages.

**FIGURE 1:**
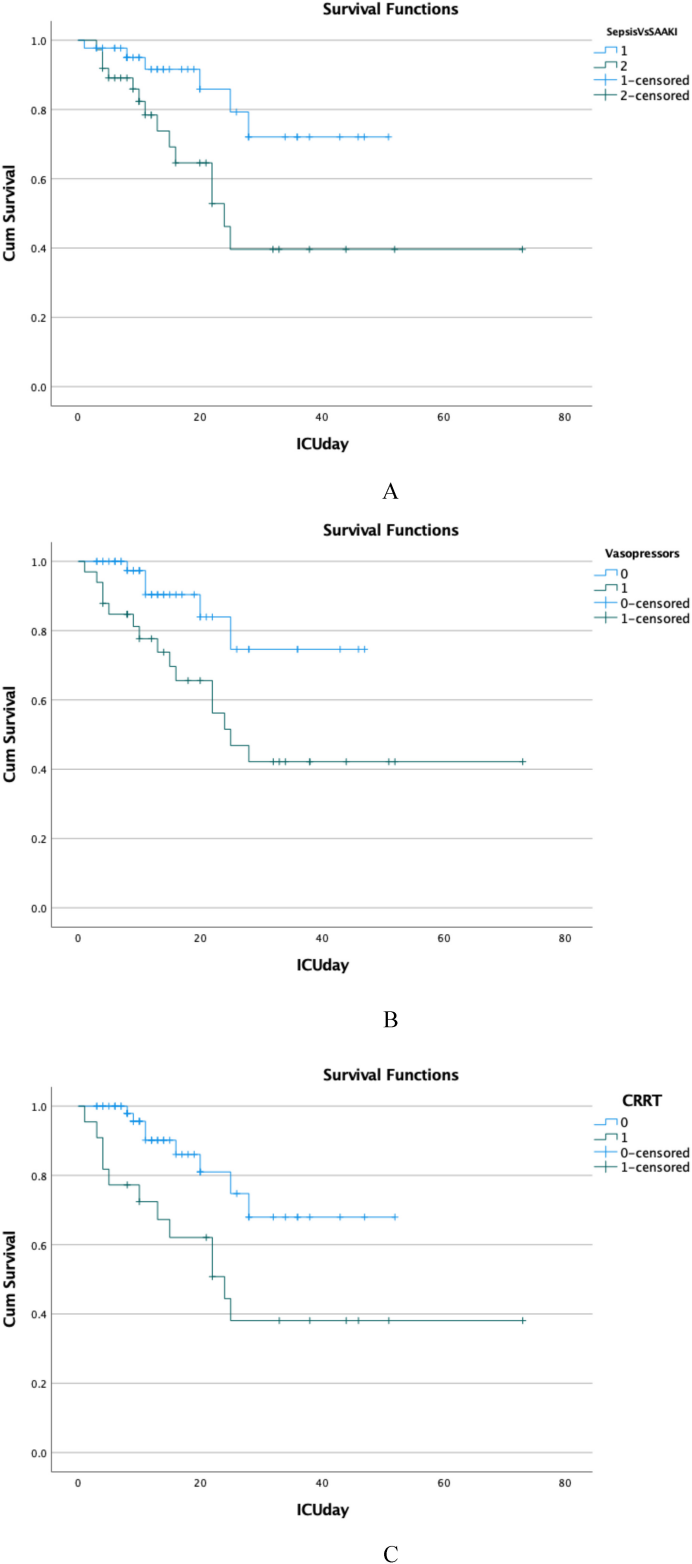
Survival curves between groups. **(A)** Sepsis vs. SA-AKI, **(B)** vasopressor vs. non-vasopressor, **(C)** CRRT vs. non-CRRT. SA-AKI, Sepsis associated Acute kidney injury; CRRT, continuous renal replacement therapy.

Significantly, there are statistical differences in the 28-day mortality rate, CRRT, and vasopressor use between the two groups. Therefore, we conducted survival curve analysis, which showed statistical differences (Figure 1A). In addition, when we categorized based on CRRT and vasopressor use, we also found that the survival curves exhibited statistical differences (Figures 1B, C).

### 3.2 Predictive value of NPS in SA-AKI

**FIGURE 2:**
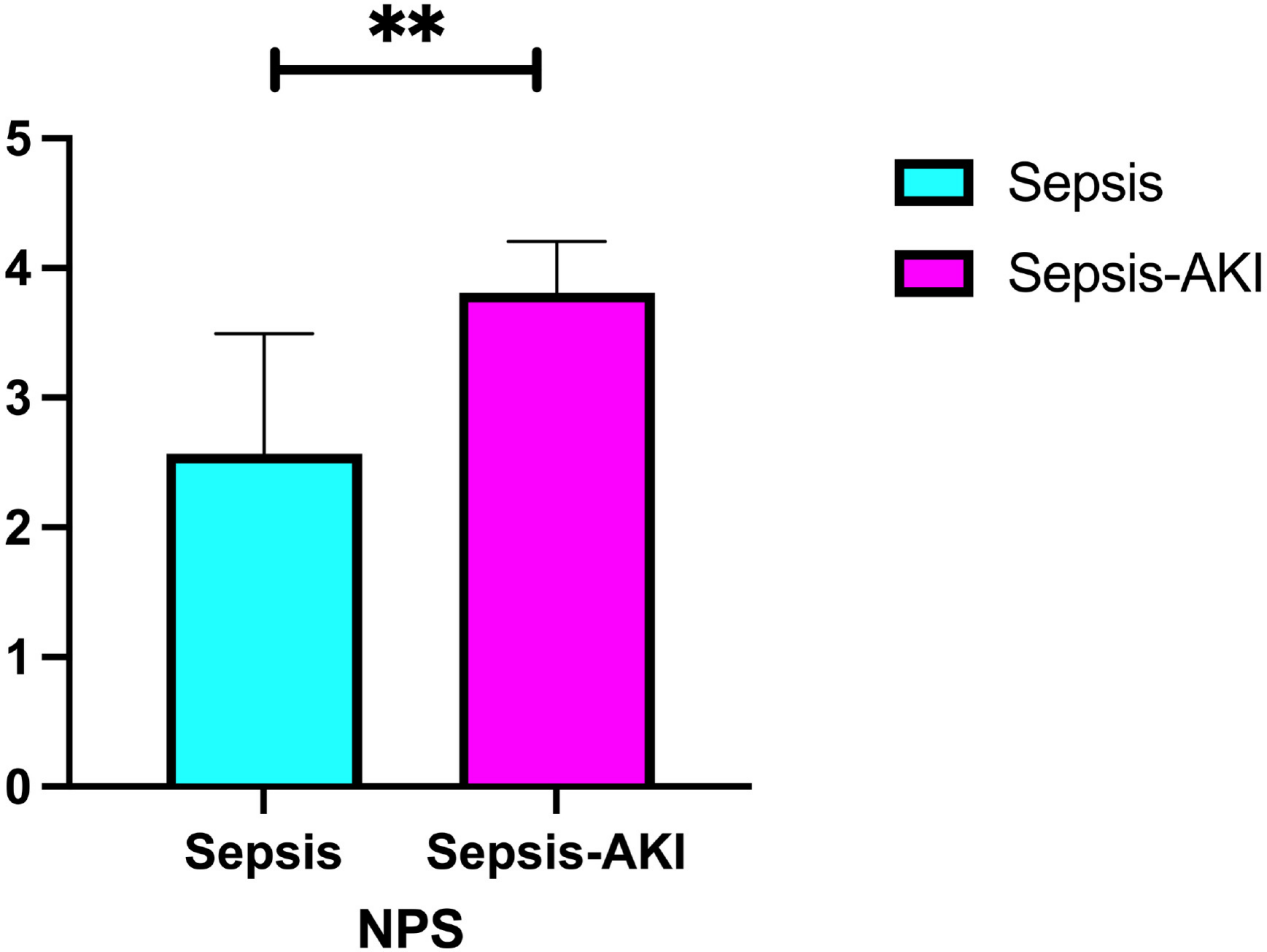
Differential expression of NPS between sepsis and SA-AKI. NPS, Naples prognosis score; SA-AKI: sepsis associated acute kidney injury. **Indicates a high level of statistical significance, corresponding to *P* < 0.001 in the study’s statistical analysis.

**FIGURE 3:**
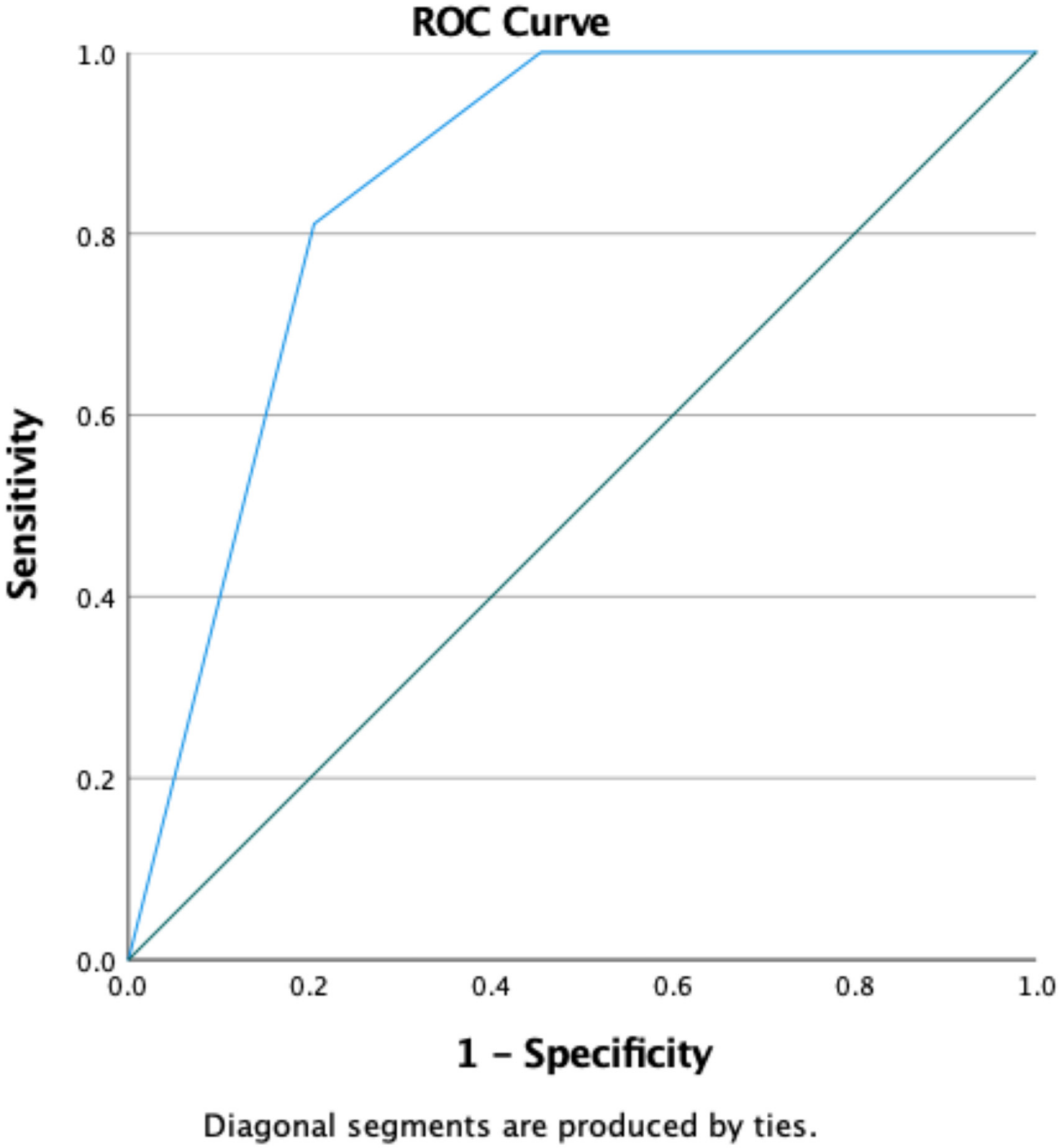
ROC curve of NPS for predicting SA-AKI. NPS, Naples prognosis score; SA-AKI, sepsis associated acute kidney injury.

Through differential analysis, we found that the NPS exhibited a statistically significant difference between the two groups (Figure 2). Our logistic regression analysis revealed that NPS is an independent risk factor between the two groups. Additionally, we plotted the prediction curve for SA-AKI and found that the AUC value for NPS in predicting SA-AKI is 0.855 (Figure 3).

### 3.3 Correlation analysis

**FIGURE 4:**
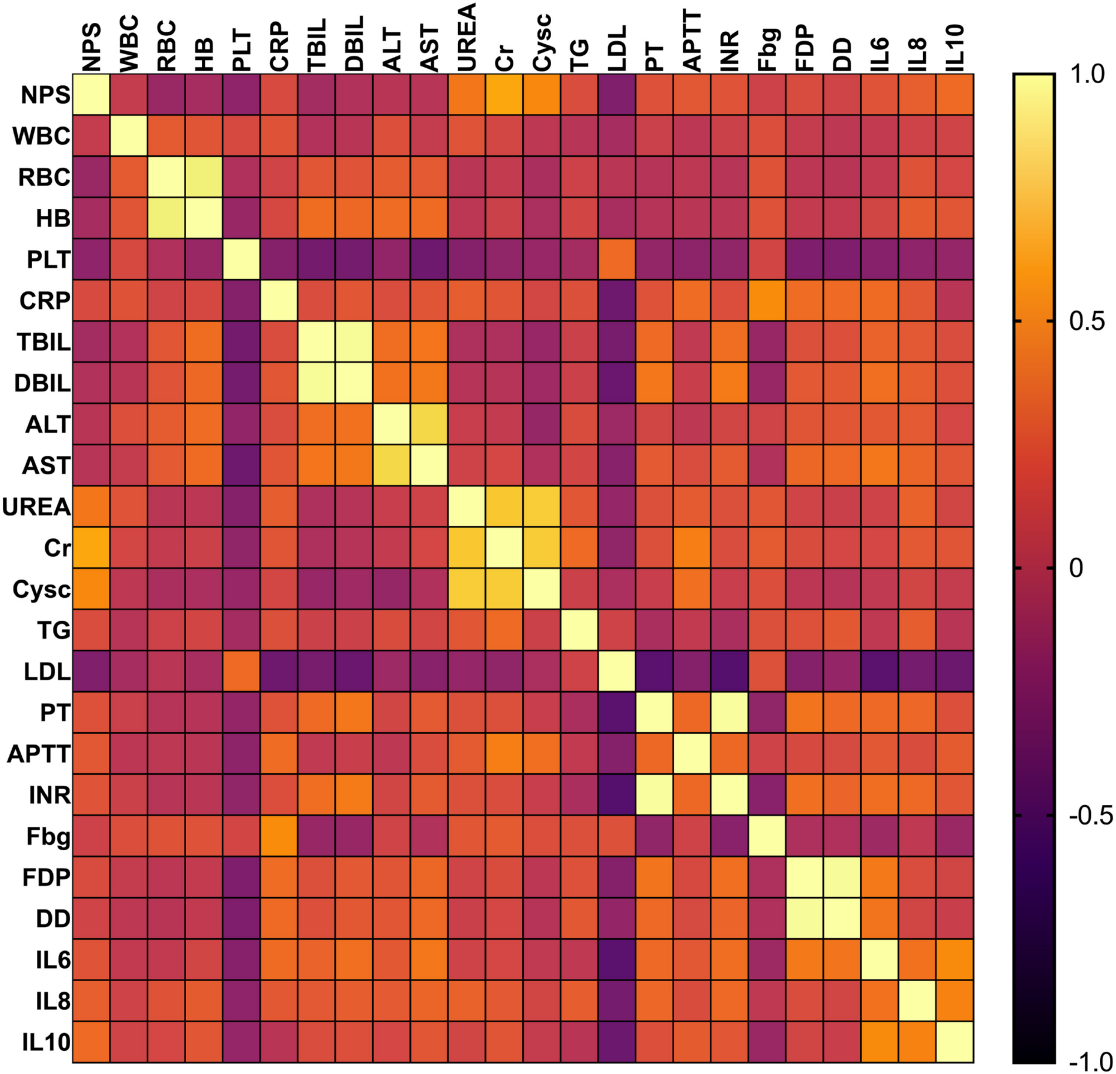
Spearman correlation heatmap.

Correlation analysis was performed on the measurement indicators, revealing positive correlation indicators with NPS: UREA (*r* = 0.394, *P* = 0.043), CR (*r* = 0.611, *P* < 0.001), CYSC (*r* = 0.475, *P* < 0.001), APTT (*r* = 0.237, *P* = 0.033), IL-8 (*r* = 0.278, *P* = 0.012), IL-10 (*r* = 0.336, *P* = 0.002). Conversely, the negative correlation indicators with NPS included: platelet (*r* = −0.225, *P* = 0.043), LDL (*r* = −0.297, *P* = 0.007) (Figure 4).

### 3.4 Multivariable logistic regression analysis for SA-AKI

Based on the statistical differences observed between the two groups and the significant findings from the single-factor analysis, we included platelet, UREA, CR, CYCS, and APTT in a multivariable logistic regression analysis. The results indicated that NPS was an independent predictor of AKI, with an OR of 11.777 (*P* < 0.001) (Table 3).

**TABLE 3:** Multivariable logistic regression analysis for SA-AKI.

Variable	B	S.E.	Wald	Df	Sig.	OR
NPS	2.466	0.589	17.504	1	<0.001	11.777
APTT	0.091	0.036	6.278	1	0.012	1.095
Constant	−12.740	2.943	18.742	1	<0.001	0.000

SA-AKI, sepsis-associated acute kidney injury; APTT, activated partial thromboplastin time.

### 3.5 Subgroup analysis (vasopressor)

**FIGURE 5:**
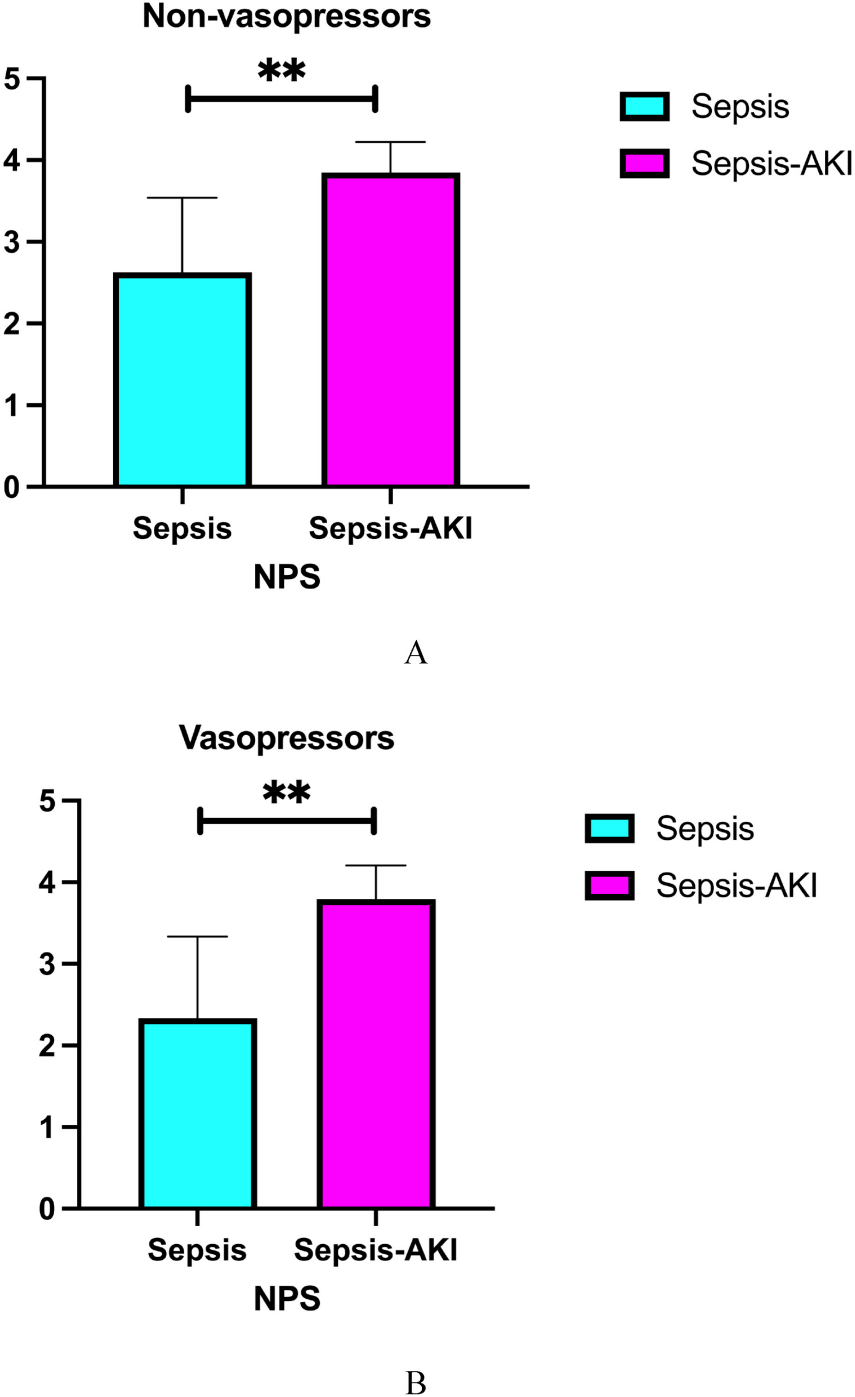
Differential expression of NPS between sepsis and SA-AKI (Subgroup analysis: Vasopressor). **(A)** Non-vasopressor, **(B)** vasopressor. NPS, Naples prognosis score; SA-AKI, sepsis associated acute kidney injury. **Indicates a high level of statistical significance, corresponding to *P* < 0.001 in the study’s statistical analysis.

We investigated the expression of NPS in the presence or absence of vasopressor medication. Our findings indicated that in the group not receiving vasoactive drugs, the NPS expression of SA-AKI was significantly increased (*P* < 0.001). In the group receiving vasoactive drugs, the NPS expression of SA-AKI also showed a significant increase (*P* < 0.001) (Figure 5).

**FIGURE 6:**
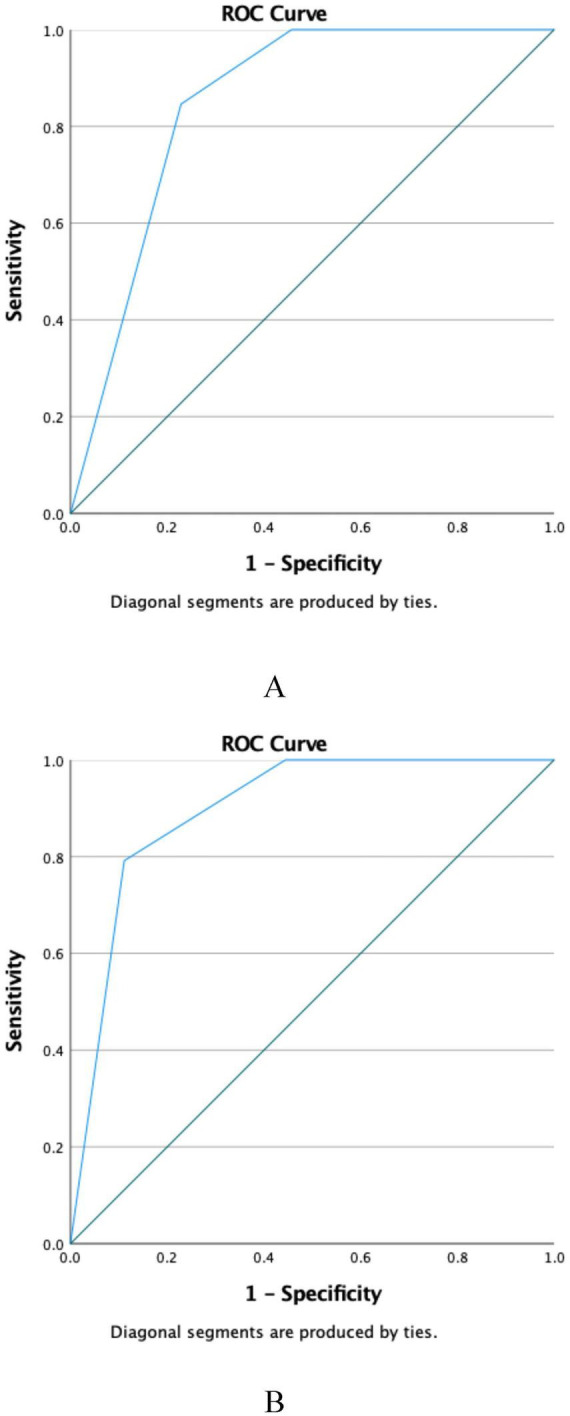
ROC curve of NPS for predicting SA-AKI (Subgroup analysis: Vasopressor). **(A)** Non-vasopressor, **(B)** vasopressor. NPS, Naples prognosis score; SA-AKI, sepsis associated acute kidney injury.

We generated the ROC curve and determined that the predicted ROC value of NPS with the use of vasopressors. Further intra-group predictive analysis was conducted. In the group not utilizing vasoactive drugs, the ROC value predicted by NPS in SA-AKI was 0.851 (*P* < 0.001) (Figure 6A). In the group receiving vasoactive drugs, the ROC value predicted by NPS in SA-AKI was 0.898 (*P* < 0.006) (Figure 6B).

### 3.6 Subgroup analysis (non-CRRT)

**FIGURE 7:**
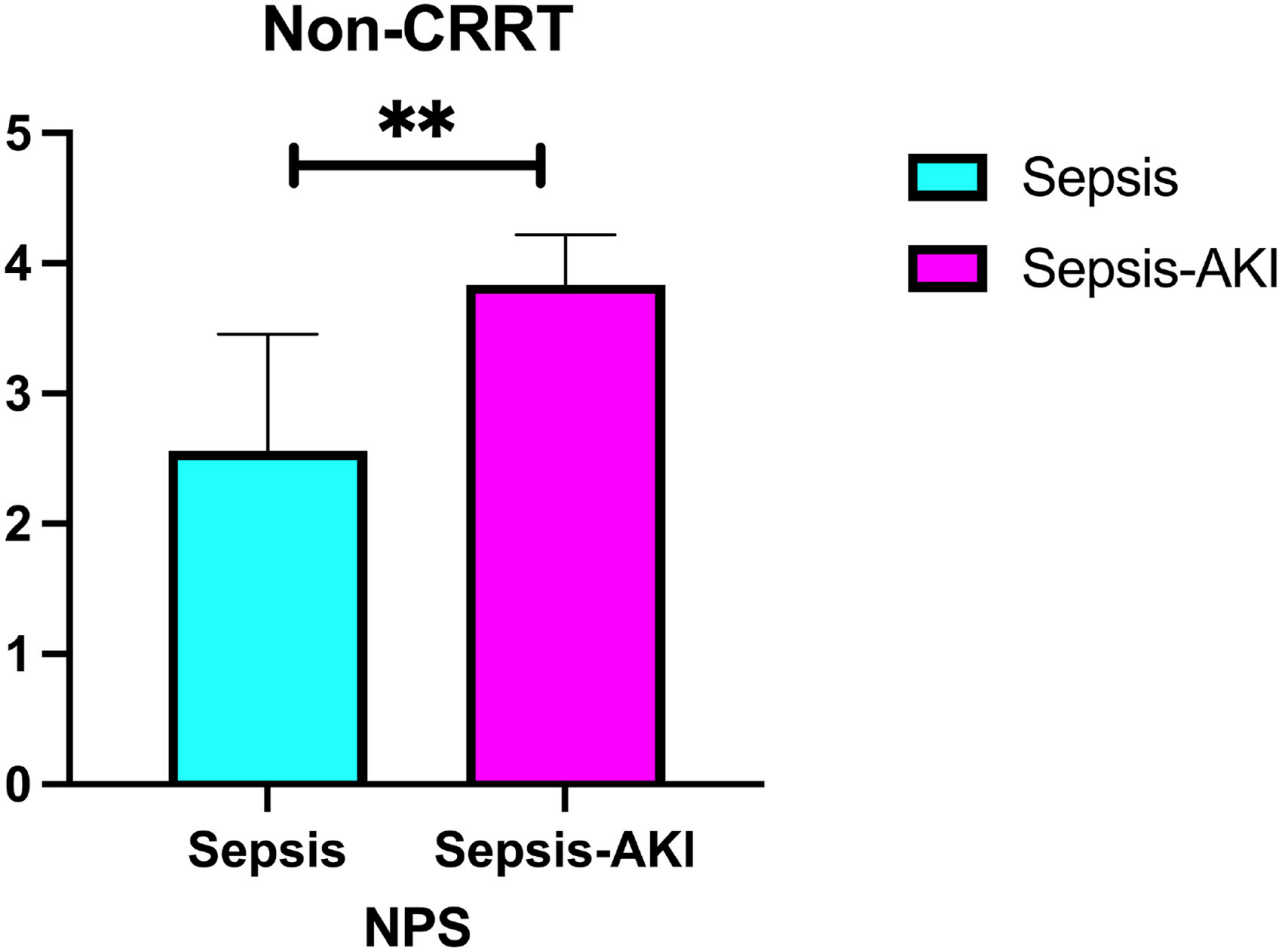
Differential expression of NPS between sepsis and SA-AKI (Subgroup analysis: Non-CRRT). NPS, Naples prognosis score; SA-AKI, sepsis associated acute kidney injury; CRRT, continuous renal replacement therapy. **Indicates a high level of statistical significance, corresponding to *P* < 0.001 in the study’s statistical analysis.

We investigate the expression of NPS with and without the application of CRRT. Intra-group analysis revealed that in the non-CRRT group, NPS expression in the SA-AKI subgroup was significantly elevated (*P* < 0.001) (Figure 7).

**FIGURE 8:**
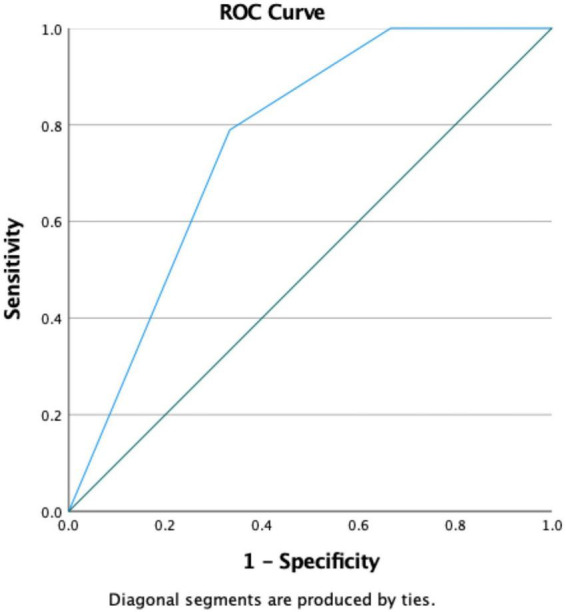
ROC curve of NPS for predicting SA-AKI (Subgroup analysis: Non-CRRT). NPS, Naples prognosis score; SA-AKI, sepsis associated acute kidney injury; CRRT, continuous renal replacement therapy.

Additional intra-group predictive analysis was conducted, showing that in the group without CRRT, the ROC value of NPS predicted by SA-AKI was 0.866 (*P* < 0.001) (Figure 8).

## 4 Discussion

This study confirms that NPS is an independent risk factor for SA-AKI (OR = 11.777, *P* < 0.001), with an area under the ROC curve (AUC) of 0.855, indicating high diagnostic efficiency. The strength of NPS lies in its integration of Inflammatory markers (NLR, LMR), nutritional (albumin), and lipid metabolic (total cholesterol) markers, reflecting the pathophysiological state of septic patients from multiple angles. Its reliance on routine laboratory parameters makes NPS cost-effective and accessible, particularly in resource-limited settings.

Mechanistically, elevated NLR reflects neutrophil activation and lymphocytic depletion, closely linked to excessive inflammation and immunosuppression in sepsis (14–16). Reduced LMR indicates monocyte dysfunction, potentially exacerbating the inflammatory storm (17–19). Hypoalbuminemia not only reflects nutritional depletion but also correlates with vascular endothelial barrier disruption, while hypocholesterolemia has been independently associated with poor outcomes in septic patients (20, 21). These factors may synergistically promote SA-AKI by enhancing renal inflammatory infiltration, microcirculatory dysfunction, and tubular epithelial cell injury (22–25).

Correlation analysis revealed positive associations of NPS with urea nitrogen UREA, CR, and CYSC, and negative associations with platelet count and LDL. These results further validate the pathophysiological basis of NPS: the correlation between NPS and renal injury markers CYSC, and UREA supports its mechanistic relevance to SA-AKI progression. Thrombocytopenia is associated with disseminated intravascular coagulation and platelet consumption in sepsis, while reduced LDL may exacerbate renal injury by impairing lipid-mediated renoprotection (26–28). Notably, the positive correlation between NPS and APTT highlights the role of coagulation dysfunction in SA-AKI. Imbalance of the coagulation-fibrinolysis system in sepsis can lead to renal microthrombosis, and NPS may indirectly predict SA-AKI by reflecting this imbalance (29, 30).

Subgroup analyses showed that NPS effectively predicted SA-AKI regardless of vasopressor use or continuous renal replacement therapy, suggesting its reliability in heterogeneous ICU populations. In the vasopressor subgroup, NPS had a higher AUC (0.898) in patients receiving vasopressors than in those not receiving them (0.851), possibly due to the more severe condition indicated by vasopressor use, enhancing NPS discriminative power. In the non-CRRT group, NPS had an AUC of 0.866, demonstrating its predictive value in patients without renal replacement therapy and supporting its use as an early screening tool.

These findings hold significant clinical relevance: NPS can assist in evaluating SA-AKI risk in septic patients requiring vasopressor support or not yet initiated on CRRT, guiding clinical decision-making. For example, patients with high NPS may warrant more intensive renal function monitoring or early intervention.

Our findings extend earlier work on prognostic scores in critical care. For instance, the SOFA score focuses on organ dysfunction but lacks metabolic parameters, while the SAPS-II emphasizes acute physiology without integrating immune-nutritional status. NPS uniquely bridges these gaps, offering a holistic risk assessment tool (31, 32).

## 5 Conclusion

This study first confirms that NPS serves as an independent predictor of SA-AKI, with its integrated inflammatory, nutritional, and metabolic markers providing new insights into SA-AKI pathophysiology. Clinically, NPS offers a simple and feasible tool to assist in early identification of high-risk SA-AKI patients, informing personalized treatment strategies. Further research is needed to explore NPS-guided interventions for improving outcomes in SA-AKI patients.

This study has several limitations: the single-center retrospective design may introduce selection bias, requiring validation in multicenter prospective studies.

## Data Availability

The raw data supporting the conclusions of this article will be made available by the authors, without undue reservation.
